# Snapchat Usage Intensity and Symptoms of Depression and Anxiety Among Adults in a Central Region of Saudi Arabia: A Cross-Sectional Study

**DOI:** 10.7759/cureus.115058

**Published:** 2026-08-23

**Authors:** Fuhaid Alqossayir

**Affiliations:** 1 Department of Family and Community Medicine, College of Medicine, Qassim University, Qassim, SAU

**Keywords:** anxiety, depression, effects of social media, epidemiology, preventive medicine, snapchat intensity score

## Abstract

Background: Social media platforms, including Snapchat, are widely used across age groups in Saudi Arabia. Evidence on how intensity of use relates to symptoms of depression and anxiety among adults in the region remains limited. This study examined the association between Snapchat usage intensity and symptoms of depression and anxiety among adults in the Qassim region of Saudi Arabia.

Methodology: A cross-sectional Arabic-language online survey was conducted between January and August 2023 in Buraydah City. Adults aged 18 to 65 years who used Snapchat at least two to four times daily were recruited from 12 schools through randomized cluster selection combined with voluntary participation among school staff and their adult family members. Snapchat usage intensity was measured using a six-item scale adapted from the Facebook Intensity Scale (range, 6-30), and symptoms of depression and anxiety were assessed using the Arabic versions of the Patient Health Questionnaire-9 (PHQ-9) and Generalized Anxiety Disorder-7 (GAD-7). Spearman's rank-order correlation and multivariable linear regression adjusted for age, marital status, and income were performed, with significance set at *P* < 0.05. Ethical approval was obtained from the Research Ethics Committee of Qassim University.

Results: A total of 323 adults participated (mean age, 32.6 ± 9.5 years; 203 (62.8%) female). The mean Snapchat intensity score was 17.78 (standard deviation (SD), 5.56), with no significant gender difference (mean difference, 0.41; 95% confidence interval (CI), -0.85 to 1.67; *P* = 0.53). Internal consistency of the adapted intensity scale was good (Cronbach's alpha 0.867). Snapchat intensity correlated weakly with anxiety scores (rho = 0.242, *P* < 0.001, 95% CI 0.130-0.348) and with depression scores (rho = 0.311, *P* < 0.001, 95% CI 0.203-0.412), accounting for 5.9% and 9.7% of variance, respectively. After adjustment for age, marital status, and income, Snapchat intensity remained associated with depression scores (*B* = 0.25, 95% CI 0.14-0.36, *P *< 0.001) and anxiety scores (*B* = 0.17, 95% CI 0.07-0.27, *P* = 0.001).

Conclusions: Snapchat usage intensity showed a small positive association with symptoms of depression and anxiety. The effect sizes were small, accounting for less than 10% of the variance in symptom scores, and the cross-sectional design precludes any inference about direction. In a sensitivity analysis, the association was attenuated by adjustment for problematic social media use.

## Introduction

Social media use is now a routine part of daily life, especially among adolescents and young adults, and this pattern has grown alongside widespread smartphone access [1,2]. In Saudi Arabia, adolescents primarily access the internet through smartphones, and social media is their most common reason for going online [2]. Snapchat is particularly relevant in this context because it is one of the most widely used platforms among young people and remains highly prevalent among Saudi young adults, with 83.5% of participants reporting use in one national survey [3]. At the same time, concern about the mental health effects of social media has increased, especially because adolescents and young adults are also age groups at elevated risk for depression and anxiety symptoms [1,4,5].

The broader literature indicates that social media use is associated with depression and anxiety, but the strength of this association depends on how use is measured. Systematic review and meta-analytic evidence suggest that problematic social media use is more consistently linked to poorer mental health than time spent alone [1,6]. A recent scoping review found that studies use many different exposure measures and that social comparison and addiction-related measures are the dimensions most consistently associated with poor mental health [7]. Time-based findings are less uniform, although heavier use still appears relevant: adolescents spending more than 3 hours per day on social media had a higher risk of later internalizing problems in a longitudinal cohort [8], and a meta-analysis during COVID-19 found that greater time spent on social media was associated with both anxiety and depressive symptoms [9].

Findings on Snapchat specifically are mixed. In a mixed-methods study, greater Snapchat intensity was associated with poorer mental health, and Snapchat behaviors such as monitoring ex-partners and using Snap Map were associated with greater romantic jealousy [10]. However, recent platform-comparison research in young adults found that greater Snapchat use was associated with fewer mental health difficulties overall, whereas TikTok and YouTube were more consistently associated with worse outcomes [5]. Saudi community data also suggest that platform effects are not uniformly harmful, as daily Snapchat use was associated with a lower depression risk, while Instagram, TikTok, Telegram, and YouTube showed the opposite pattern [11]. More generally, problematic use across multiple platforms, including Snapchat, is associated with greater depression and loneliness, and youth with problematic use report more depressive symptoms, anxiety, suicidal thoughts, and poorer well-being than non-problematic users [4,12].

Evidence from Saudi Arabia further supports the relevance of studying intensive engagement rather than exposure alone. In Saudi young adults, social media addiction was common and was associated with lower self-esteem and greater loneliness [3]. Experimental evidence also suggests that reducing social media use can improve mental health: a randomized controlled trial found that a one-week break from social media improved well-being and reduced symptoms of depression and anxiety [13], although that intervention concerned social media generally rather than any single platform.

Given the mixed Snapchat-specific findings, the limitations of duration-based measures, and the high prevalence of Snapchat use in Saudi Arabia, the purpose of this study was to determine whether Snapchat use intensity is associated with symptoms of depression and anxiety among adults in Buraydah city. The primary outcomes were the PHQ-9 total score and the GAD-7 total score, both analyzed as continuous variables. The hypothesis was that higher Snapchat intensity scores would be associated with higher symptom scores, and whether any observed associations persisted after adjustment for demographic characteristics was examined. The study was exploratory with respect to mechanisms, as no mediating variables were measured.

## Materials and methods

Study design and setting

This analysis forms part of a larger project examining social media use and mental health in the adult population of Buraydah, Qassim region, Saudi Arabia. The overall project, its recruitment protocol, and its findings on general patterns of social media use are reported elsewhere [14].

A descriptive, cross-sectional design was used. Data were collected in a single period between January and August 2023, with the study team visiting the participating schools during the early months of that period to enrol staff and invite onward distribution to their adult family members. The present analysis is restricted to those participants in the larger project who met the Snapchat use criterion described below, and is therefore a subgroup analysis of the project sample rather than an independent study.

The purpose of the present analysis was to examine the relationship between Snapchat usage intensity and symptoms of depression and anxiety. Snapchat was selected because it is among the most widely used platforms in Saudi Arabia and because platform-specific evidence, as distinct from evidence on aggregate social media use, remains limited in this setting.

Study population and sampling procedure

Residents of Buraydah aged between 18 and 65 years were eligible. For the present analysis, eligibility was further restricted to participants who reported using Snapchat at least two to four times per day, a criterion adopted to ensure that the intensity scale was completed by habitual users for whom the items were meaningful. A total of 323 participants met these criteria. Additional inclusion requirements were proficiency in Arabic and the provision of informed electronic consent.

Recruitment used a multistage procedure. Buraydah was divided into three geographical zones (north, central, and south). Within each zone, four schools were selected at random from the list of schools, comprising two male-only and two female-only schools, giving 12 recruitment clusters. School administrators distributed the survey link to teaching and administrative staff, who were invited to forward it to their adult family members.

Participation at the individual level was voluntary and was not randomly sampled. The procedure is therefore best described as randomized selection of clusters combined with voluntary response sampling within those clusters. Because participation depended on staff networks, the resulting sample cannot be regarded as representative of the adult population of Buraydah or of the Qassim region, and this is addressed in the Limitations.

The sample size for the larger project was calculated using G*Power version 3.1.9.7, assuming an effect size of 0.25, statistical power of 80%, and an alpha of 0.05, giving a required sample of 506 participants; this target was met. No separate a priori calculation was performed for the present subgroup, whose size was determined by the number of project participants meeting the Snapchat use criterion. With 323 participants, this analysis had 80% power to detect a correlation of 0.155 or greater at a two-tailed alpha of 0.05, which is smaller than both observed coefficients.

A conventional response rate cannot be calculated because the survey link was distributed onward by school administrators and by staff to their family members, so the number of adults who received the invitation is unknown. Individuals who declined consent were excluded before completing the questionnaire.

Data collection tool and measures

A structured, self-administered online questionnaire was developed in Arabic and comprised four sections.

The first section gathered demographic and background information: age, gender, marital status, education level, employment status, and income.

The second section characterized social media use. Participants reported the total number of minutes spent on social media per day, the number of platforms used from a list of seven (WhatsApp, Twitter, Snapchat, Instagram, TikTok, Facebook, and YouTube), and the frequency of visits to each platform. Visit frequency was scored as 0 for "I do not use this platform" and "less than once a week", 1.5 for one to two times per week, 4.5 for three to six times per week, 7 for about once a day, 21 for two to four times a day, and 35 for five or more times a day, following the scoring used in the parent project [14]. This section also included a six-item measure of problematic social media use adapted from the Bergen Facebook Addiction Scale, scored from 0 to 24, with scores of 12 or above classified as high problematic use, again following [14].

The third section measured Snapchat usage intensity using a six-item scale adapted from the Facebook Intensity Scale developed by Ellison et al. [15]. The scale measures emotional connectedness to the platform and its behavioral integration into daily routines. Each item was rated on a five-point Likert scale, giving a total score from 6 to 30, with higher scores reflecting more intense use. The original Facebook Intensity Scale has been validated; however, this validation does not establish the validity of the Snapchat adaptation, and no published validation of the adapted scale in an Arabic-speaking population exists [16].

A score above 19 was used to define high-intensity use. This threshold was derived from the distribution of intensity scores in the present sample, in which the mean was 17.78 and the median 17, and it classified 122 participants (37.8%) as high-intensity users and 201 (62.2%) as low-intensity users. No previously published cutoff exists for the Snapchat adaptation of the scale. The threshold is therefore sample-specific and should not be applied to other populations.

The fourth section assessed mental health. Depressive symptoms were measured using the Patient Health Questionnaire-9 (PHQ-9) and anxiety symptoms using the Generalized Anxiety Disorder 7-item scale (GAD-7). Arabic versions of both instruments were used, on the basis of the adaptation and validation reported by Sawaya et al. [17]. That validation was conducted in a Lebanese psychiatric outpatient sample, and psychometric performance in a Saudi general adult population has not been separately established. Both instruments screen for symptom severity and do not establish a clinical diagnosis.

Validity and reliability

The content validity of the instrument was reviewed by a panel of three academic experts in psychology and public health. A pilot study with 20 participants from the target population assessed clarity, readability, and internal consistency, and item wording was refined for clarity and cultural appropriateness on the basis of the feedback received.

Internal consistency was calculated in the final sample for all four scales. Cronbach's alpha was 0.867 (95% CI 0.843-0.888) for the adapted Snapchat Intensity Scale, 0.891 for the PHQ-9, 0.892 for the GAD-7, and 0.712 for the problematic social media use scale. McDonald's omega for the Snapchat Intensity Scale was 0.870. Item-rest correlations for the intensity scale ranged from 0.565 to 0.792, and removal of no item would have improved alpha. Factor structure and convergent validity of the adapted scale were not examined.

Statistical analysis

Analyses were performed using IBM SPSS Statistics (version 27; IBM Corp., Armonk, NY) and JASP (Version 0.19.3; JASP Team, University of Amsterdam, Amsterdam, The Netherlands).

Descriptive statistics were computed for all demographic and categorical variables. Normality was assessed using the Shapiro-Wilk test. Independent-samples t-tests compared mean Snapchat intensity scores between gender groups, with Levene's test for equality of variances, Cohen's d as the effect size, and the Mann-Whitney U test reported alongside where the normality assumption was not satisfied.

Spearman's rank-order correlation was used to examine the relationship between Snapchat intensity scores and PHQ-9 and GAD-7 scores, owing to the non-normal distribution of the mental health measures.

Multivariable linear regression models were fitted with PHQ-9 and GAD-7 total scores as the dependent variables and Snapchat intensity as the exposure of interest. Covariates were selected on the criterion that a confounder must be associated with both the exposure and the outcome. Age, marital status, and income met this criterion and were entered. Gender and education level were associated with neither Snapchat intensity nor symptom scores and were not entered; a sensitivity analysis confirmed that their inclusion did not materially alter the coefficient for Snapchat intensity. Income was collapsed to four categories, with the top two bands combined because only 11 participants reported income above 20,000 SAR.

A further sensitivity analysis added problematic social media use to both models. This variable was not included in the primary models because it was analyzed in the parent project [14] and because it is plausibly an intermediate variable on the pathway between intense platform engagement and symptoms rather than a confounder of that relationship.

A two-tailed *P*-value less than 0.05 was considered statistically significant.

Ethical considerations

Ethical approval was obtained from the Research Ethics Committee of Qassim University (Approval No. 22-15-07). All participants provided informed electronic consent after reading a brief description of the study objectives, confidentiality assurances, and the voluntary nature of participation. To ensure participant privacy, all data were anonymized and stored in a secure environment with restricted access.

## Results

The final study cohort comprised 323 participants, whose characteristics are shown in Table 1. Of these, 203 (62.8%) were female, and the mean age was 32.6 years (SD, 9.5; median, 33; interquartile range (IQR), 26-40). Regarding marital status, 202 participants (62.5%) were married, and 121 (37.5%) were unmarried. Educational attainment was high, with 218 participants (67.5%) holding a university degree or above. Teachers accounted for 125 participants (38.6%), and a further 72 (22.3%) were employed in non-teaching roles.

**Table 1:** Basic characteristics of the study population (N = 323). Values are *n* (%) unless otherwise indicated. Unmarried comprises 111 (34.4%) single, 7 (2.2%) divorced, and 3 (0.9%) widowed participants. Employed, non-teaching comprises 68 (21.1%) employed and 4 (1.2%) retired participants; teachers were coded separately.

Variable	Category	*n* (%)
Age (years)	Mean ± SD	32.6 ± 9.5
	Median (IQR)	33 (26-40)
	Range	18-57
	18-24	69 (21.4)
	25-34	116 (35.8)
	35-44	100 (31.0)
	45 and above	38 (11.8)
Gender	Male	120 (37.2)
	Female	203 (62.8)
Nationality	Saudi	319 (98.8)
	Non-Saudi	4 (1.2)
Education level	Primary school or less	2 (0.6)
	Middle/high school	81 (25.1)
	Diploma	22 (6.8)
	University degree and above	218 (67.5)
Marital status	Married	202 (62.5)
	Unmarried	121 (37.5)
Occupation	Unemployed	28 (8.7)
	Housewife	39 (12.1)
	Student	59 (18.3)
	Employed, non-teaching	72 (22.3)
	Teacher	125 (38.6)
Income (SAR/month)	Less than 1500	70 (21.7)
	1500-4999	80 (24.8)
	5000-9999	64 (19.8)
	10,000-20,000	98 (30.3)
	More than 20,000	11 (3.4)

Relative to the full project sample reported in [14], the present Snapchat-using subgroup was slightly younger (32.6 versus 34.3 years), included a higher proportion of women (62.8% versus 56.4%), and reported heavier overall social media use (202 versus 181 minutes per day) and a higher mean problematic use score (8.4 versus 7.9). These differences are consistent with the restriction of the present analysis to participants using Snapchat at least twice daily and indicate that the subgroup is not simply a random fraction of the project sample.

Social media usage patterns

As shown in Table 2, 279 participants (86.3%) reported using social media for more than three years, indicating sustained exposure. Only 27 (8.4%) had started within the past year, and 17 (5.3%) had used social media for one to three years. Regarding notification settings, 164 participants (50.8%) used both sound and pop-up text notifications, 104 (32.2%) opened the app to check for updates, and 55 (17.0%) received numerical badge notifications only.

**Table 2:** Social media use among Snapchat users (N = 323). Values are *n* (%) unless otherwise indicated. Operational definitions: high daily duration, more than 60 minutes per day; high weekly visit frequency, more than 74 visits per week summed across all seven platforms; high platform use, five or more platforms; high problematic social media use, a score of 12 or above on the six-item scale (range 0-24). Weekly visit frequency was scored as 0 for non-use or less than weekly use, 1.5 for one to two times weekly, 4.5 for three to six times weekly, 7 for about once daily, 21 for two to four times daily, and 35 for five or more times daily, summed across platforms. IQR, interquartile range; SD, standard deviation

Variable	Category	*n* (%)
Duration of social media use	Less than 1 year	27 (8.4)
	1 to less than 3 years	17 (5.3)
	More than 3 years	279 (86.3)
Notification type used	Sound and pop-up text	164 (50.8)
	App badge count	55 (17.0)
	None (requires opening app)	104 (32.2)
Minutes on social media per day	Mean ± SD	202.2 ± 224.0
	Median (IQR)	120 (50-300)
	Low duration	108 (33.4)
	High duration	215 (66.6)
Weekly social media visits	Mean ± SD	95.4 ± 38.5
	Median	92
	Low visits	102 (31.6)
	High visits	221 (68.4)
Problematic social media use	Mean ± SD	8.43 ± 4.10
	Low	252 (78.0)
	High	71 (22.0)
Number of platforms used	Mean ± SD	5.20 ± 1.07
	2	4 (1.2)
	3	16 (5.0)
	4	60 (18.6)
	5	100 (31.0)
	6	116 (35.8)
	7	27 (8.4)

High daily social media use was reported by 215 participants (66.6%) and high weekly visit frequency by 221 (68.4%). The mean daily duration was 202 minutes (SD 178, median 150), and the mean weekly visit score across all platforms was 95.4 (SD 40.6). Most participants (243, 75.2%) used five or more platforms, with six the most common number (116, 35.8%). High problematic social media use was reported by 71 participants (22.0%). The thresholds defining each dichotomized variable are given in the footnote to Table 2.

Mental health status

The mental health assessment, detailed in Table 3, showed that 161 participants (49.8%) had minimal depressive symptoms, with the remainder reporting mild (85, 26.3%), moderate (45, 13.9%), moderately severe (22, 6.8%), and severe (10, 3.2%) symptoms. For anxiety, 169 participants (52.3%) reported minimal symptoms, with the remainder reporting mild (81, 25.1%), moderate (49, 15.2%), and severe (24, 7.4%) symptoms.

**Table 3:** Depression and anxiety symptom severity among study participants. Values are *n* (%) unless otherwise indicated. PHQ-9 severity bands: minimal 0-4, mild 5-9, moderate 10-14, moderately severe 15-19, severe 20-27. GAD-7 severity bands: minimal 0-4, mild 5-9, moderate 10-14, severe 15-21. A score of 10 or above on either instrument is a commonly used threshold indicating at least moderate symptom severity. GAD-7, Generalized Anxiety Disorder 7-item scale; PHQ-9, Patient Health Questionnaire-9; SD, standard deviation

Instrument	Category	*n* (%)
PHQ-9 depression	Mean ± SD	6.01 ± 5.70
	Minimal	161 (49.8)
	Mild	85 (26.3)
	Moderate	45 (13.9)
	Moderately severe	22 (6.8)
	Severe	10 (3.2)
	Score 10 or above	77 (23.8)
GAD-7 anxiety	Mean ± SD	5.47 ± 5.08
	Minimal	169 (52.3)
	Mild	81 (25.1)
	Moderate	49 (15.2)
	Severe	24 (7.4)
	Score 10 or above	73 (22.6)

Applying commonly used screening thresholds, 77 participants (23.8%) scored 10 or above on the PHQ-9 and 73 (22.6%) scored 10 or above on the GAD-7, indicating at least moderate symptom severity. Both instruments screen for symptom severity and do not establish a clinical diagnosis.

Snapchat intensity score by gender

Table 4 compares Snapchat intensity scores between genders. Men scored slightly higher on average (*M* = 18.03, SD = 5.62) than women (*M* = 17.63, SD = 5.53), with a mean difference of 0.41 (95% CI, -0.85 to 1.67). This difference was not statistically significant, *t*(321) = 0.64, *P* = 0.53, and the effect size was negligible (Cohen's *d* = 0.07, 95% CI, -0.15 to 0.30). Levene's test indicated that equal variances could be assumed (*W* = 0.05, *P* = 0.83).

**Table 4:** Comparison of Snapchat usage intensity scores by gender. *Independent-samples t-test. Levene's test indicated equality of variances (*W* = 0.05, *P* = 0.83); the Welch correction gave the same result (*t* = 0.63, *P* = 0.53). Shapiro-Wilk indicated a modest departure from normality in the whole sample (*W* = 0.99, *P* = 0.004), though not within either gender group (males: *W* = 0.98, *P* = 0.058; females: *W* = 0.99, *P* = 0.050). The Mann-Whitney U test gave the same conclusion (*U* = 12551.5, *P* = 0.65). CI, confidence interval; SD, standard deviation

Variable	*n* (%)	Mean (SD)	Median	Range	Mean difference (male-female)	95% CI for mean difference	*P*-value*	Cohen's *d* (95% CI)
Male	120 (37.2)	18.03 (5.62)	17	6-30	0.41	-0.85 to 1.67	0.53	0.07 (-0.15 to 0.30)
Female	203 (62.8)	17.63 (5.53)	18	6-30				
All	323 (100)	17.78 (5.56)	17	6-30				

The Shapiro-Wilk test indicated a modest departure from normality in the whole sample (*W* = 0.99, *P* = 0.004), although not within either gender group considered separately. The Mann-Whitney U test was therefore performed alongside the t-test and yielded the same conclusion (*U* = 12551.5, *P* = 0.65). The median score was 17 among men and 18 among women, a pattern that differs slightly from that of the means and reflects the shape of the distribution rather than a difference in central tendency between the groups.

Correlation between Snapchat use and mental health

Table 5 shows the correlations between Snapchat usage intensity and mental health outcomes. A weak positive correlation was found between Snapchat intensity and GAD-7 anxiety scores (Spearman's rho = 0.242, *P* = 1.07 × 10^-5^, 95% CI 0.130-0.348). A somewhat stronger but still weak correlation was found with PHQ-9 depression scores (rho = 0.311, *P* = 1.10 × 10^-8^, 95% CI 0.203-0.412).

**Table 5:** Correlation between Snapchat usage intensity and symptom scores. *Spearman's rank-order correlation. Both coefficients represent weak associations, accounting for less than 10% of the variance in symptom scores. Estimates are unadjusted; adjusted models are presented in Table 6. CI, confidence interval; GAD-7, Generalized Anxiety Disorder 7-item scale; PHQ-9, Patient Health Questionnaire-9

Variable	Spearman's rho*	*P*-value	Lower 95% CI	Upper 95% CI	Shared variance
Snapchat intensity and GAD-7 anxiety score	0.242	<0.001	0.137	0.342	5.9%
Snapchat intensity and PHQ-9 depression score	0.311	<0.001	0.209	0.407	9.7%

These coefficients correspond to 5.9% and 9.7% of shared variance, respectively. More than 90% of the variance in symptom scores was therefore not accounted for by Snapchat intensity.

**Figure 1:**
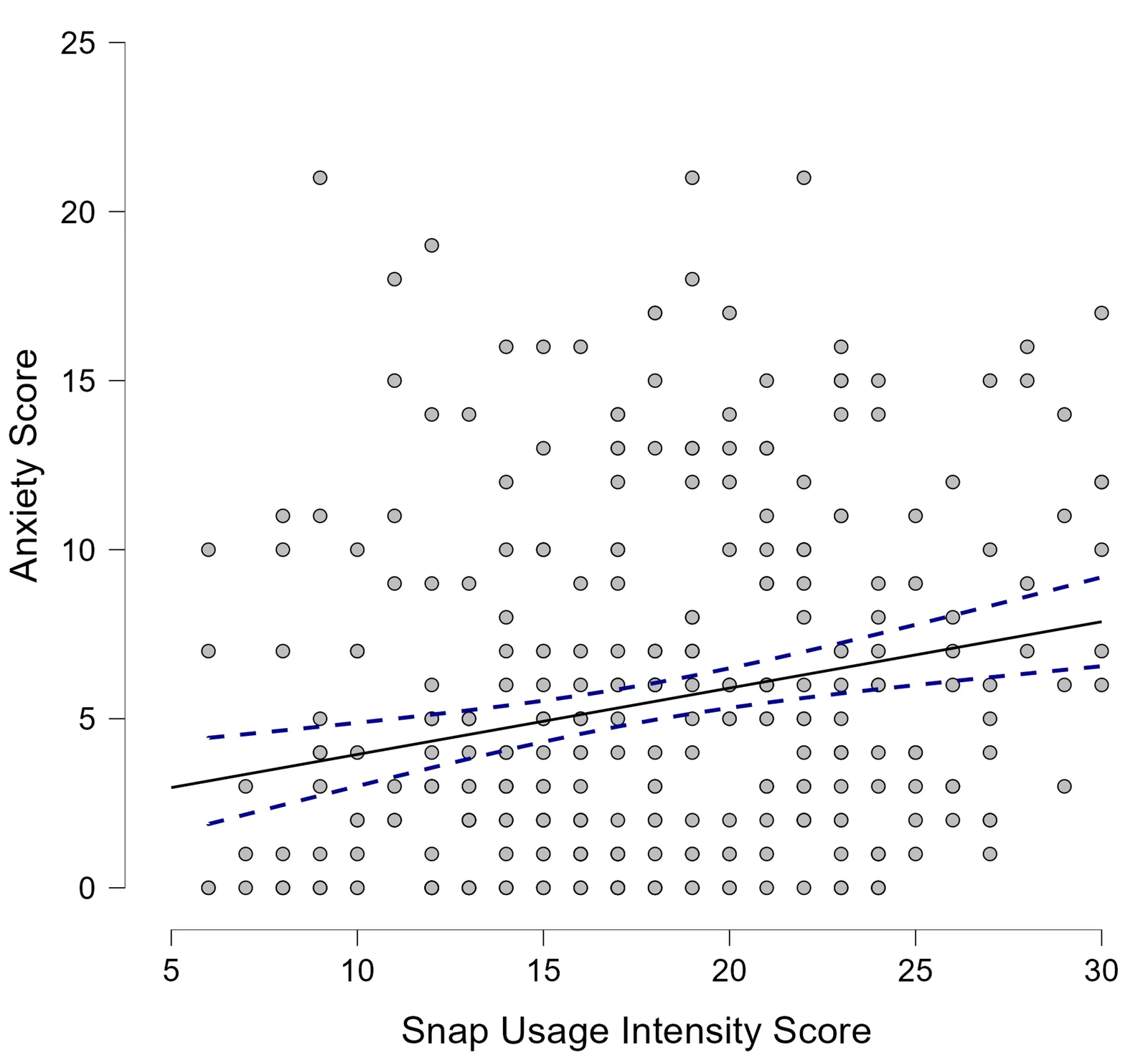
Association between Snapchat usage intensity score and GAD-7 anxiety score. The reported coefficient was Spearman's rho = 0.242, *P* < 0.001, 95% CI, 0.130-0.348. GAD-7, Generalized Anxiety Disorder 7-item scale; CI, confidence interval

**Figure 2:**
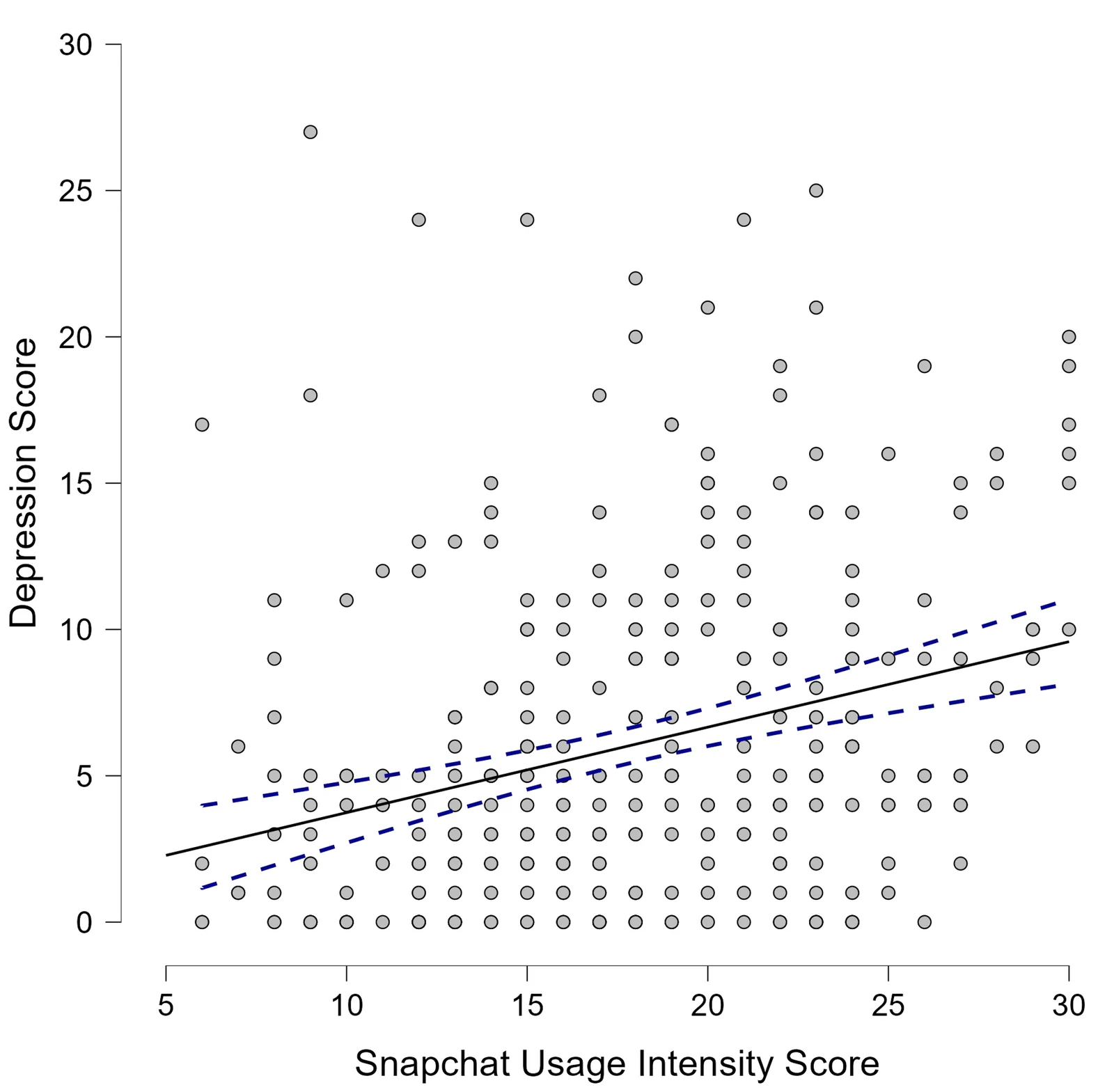
Association between Snapchat usage intensity score and PHQ-9 depression score. The reported coefficient is Spearman's rho = 0.311, *P* < 0.001, 95% CI, 0.203-0.412. PHQ-9, Patient Health Questionnaire-9; CI, confidence interval

The relationships identified in Table 5 are further illustrated in Figures 1-2. Figure 1 displays a scatter plot of Snapchat intensity scores versus anxiety scores, highlighting a mild upward trend, reflective of the positive correlation. In contrast, Figure 2 shows a clearer upward trend between Snapchat intensity scores and depression scores, visually supporting the relatively stronger correlation found between Snapchat use and depressive symptoms.

These findings collectively suggest that while Snapchat use is widespread and intense among the participants, higher emotional and habitual engagement with the platform may be weakly to moderately associated with elevated symptoms of anxiety and depression.

Multivariable analysis

Table 6 presents the multivariable linear regression models. After adjustment for age, marital status, and income, Snapchat usage intensity remained associated with depression scores (*B* = 0.25, 95% CI 0.14-0.36, standardized beta = 0.24, *P* < 0.001) and with anxiety scores (*B* = 0.17, 95% CI 0.07-0.27, standardized beta = 0.18, *P* = 0.001). The models accounted for 12.5% and 6.9% of the variance in depression and anxiety scores, respectively, *F*(6, 316) = 7.51, *P* < 0.001, and *F*(6, 316) = 3.89, *P* < 0.001.

**Table 6:** Multivariable linear regression of depression and anxiety symptom scores on Snapchat usage intensity. Adjusted for age, marital status, and income. Reference categories: married; income below 1,500 SAR. PHQ-9, Patient Health Questionnaire-9; GAD-7, Generalized Anxiety Disorder 7-item scale; SE, standard error; CI, confidence interval

Predictor	*B*	SE	Beta	*t*	*P*	95% CI
PHQ-9 depression score: *R*² = 0.125, adjusted *R*² = 0.108, *F*(6, 316) = 7.51, *P* < 0.001						
Snapchat intensity (per point)	0.250	0.056	0.244	4.47	<0.001	0.140 to 0.361
Age (per year)	-0.036	0.047	-0.061	-0.78	0.436	-0.128 to 0.055
Unmarried vs. married	2.039	0.830	-	2.46	0.015	0.406 to 3.672
Income 1,500-4,999 vs. <1,500	-0.374	0.933	-	-0.40	0.689	-2.211 to 1.462
Income 5,000-9,999 vs. <1,500	0.617	1.021	-	0.61	0.546	-1.391 to 2.625
Income ≥10,000 vs. <1,500	0.208	1.022	-	0.20	0.839	-1.803 to 2.218
GAD-7 anxiety score: *R*² = 0.069, adjusted *R*² = 0.051, F(6, 316) = 3.89, *P* < 0.001						
Snapchat intensity (per point)	0.166	0.051	0.182	3.23	0.001	0.065 to 0.267
Age (per year)	-0.026	0.043	-0.049	-0.61	0.540	-0.111 to 0.058
Unmarried vs. married	1.172	0.762	-	1.54	0.125	-0.328 to 2.671
Income 1,500-4,999 vs. <1,500	0.105	0.857	-	0.12	0.903	-1.582 to 1.791
Income 5,000-9,999 vs. <1.500	-0.064	0.937	-	-0.07	0.945	-1.909 to 1.780
Income ≥10,000 vs. <1,500	-0.087	0.938	-	-0.09	0.927	-1.933 to 1.760

Each one-point increase in Snapchat intensity score corresponded to a 0.25-point higher PHQ-9 score and a 0.17-point higher GAD-7 score. Across the observed range of the intensity scale, from 6 to 30, this corresponds to a difference of approximately 6.0 PHQ-9 points and 4.0 GAD-7 points between the lowest- and highest-scoring participants.

Among the covariates, unmarried status was independently associated with higher depression scores (*B* = 2.04, 95% CI 0.41-3.67, *P* = 0.015). Neither age nor income was independently associated with either outcome after adjustment.

In a sensitivity analysis that additionally adjusted for problematic social media use, the association between Snapchat intensity and depression scores was attenuated and was no longer statistically significant (*B* = 0.09, 95% CI -0.02 to 0.20, *P* = 0.12), as was the association with anxiety scores (*B* = 0.02, 95% CI -0.08 to 0.12, *P* = 0.74). Problematic social media use was independently associated with both outcomes (*P* < 0.001), and the addition of this variable increased the explained variance to 26.8% for depression and 21.8% for anxiety.

## Discussion

This study investigated the association between the intensity of Snapchat use and symptoms of depression and anxiety among a diverse adult sample in the Qassim region of Saudi Arabia. The results offer important insights into how behavioral and emotional engagement with this visual-based social media platform may relate to mental health symptoms.

The findings showed statistically significant but weak positive correlations between emotional and behavioral engagement with Snapchat and symptoms of depression and anxiety, and these associations persisted after adjustment for age, marital status, and income. The direction of these findings is consistent with reviews reporting associations between problematic or intensive social media use and poorer mental health [18,19], although that evidence derives largely from adolescent samples and the associations reported by Huang [19] were themselves weak.

The magnitude of association was small. Snapchat intensity accounted for 5.9% of the variance in anxiety scores and 9.7% of the variance in depression scores, leaving more than 90% unexplained in each case. Statistical significance in a sample of this size should not be read as clinical significance, and these results do not identify Snapchat intensity as an important determinant of symptoms at the individual level.

Because exposure and outcome were measured at a single time point, the direction of the observed associations cannot be determined. It is equally consistent with these data that adults with more depressive or anxious symptoms engage more intensively with Snapchat, or that a third factor accounts for both. Reverse causation and reciprocal influence remain plausible explanations.

The present finding differs from that of BinDhim et al. [11], who reported that daily Snapchat use was associated with a lower risk of depression in a Saudi community sample. Several differences may account for the divergence. The exposure definitions are not equivalent: [11] classified participants according to whether they used the platform daily, whereas the present study measured emotional and behavioral intensity on a continuous scale among participants who already used Snapchat at least twice daily. A person may open an application daily without high emotional investment, and the two measures therefore capture different constructs. The sampling frames also differ, since [11] drew on a national panel, whereas the present sample was recruited through schools and was more highly educated. Finally, [11] adjusted for a broader set of lifestyle covariates. The two findings are not directly comparable, and the discrepancy illustrates the sensitivity of platform-level conclusions to how exposure is operationalized.

None of the mechanisms discussed in the following paragraph were measured in the present study. Fear of missing out, social comparison, compulsive checking, sleep quality, and emotional regulation were not assessed, and the explanations offered are hypotheses for future testing rather than interpretations supported by these data.

One proposed mechanism is that real-time, visual platforms may promote fear of missing out and increase emotional arousal, both of which have been reported to be associated with heightened anxiety in social networking research more generally [20]. The ephemeral design of Snapchat has been proposed as a further contributor, because content expires and users may check the application frequently to avoid missing it [10,21,22]. With respect to depressive symptoms, emotionally intensive engagement with visual platforms has been reported to be associated with poorer well-being [23-25]. Problematic smartphone use has also been reported to be associated with symptoms of depression and anxiety [26,27].

These findings are consistent with the broader literature suggesting that emotionally involved or problematic patterns of social media use may be more clinically relevant than duration alone [28,29]. In the present adjusted models, daily duration of social media use was not associated with either outcome. This observation derives from a cross-sectional analysis and should not be read as establishing the relative importance of the two constructs.

Snapchat remains a predominant platform in Saudi Arabia [3,11], although the evidence cited concerns young adults whereas the present sample spans a broader adult age range with a mean age of 32.6 years. The results should therefore be read as describing habitual adult Snapchat users in one city rather than as characterizing Saudi young adults generally.

This study has several limitations. Recruitment was centered on Buraydah city and conducted through schools rather than through an openly distributed online survey. Although school clusters were selected at random, individual participation was voluntary and confined to school staff and their adult family members. Teachers accounted for 38.6% of the sample and 67.5% held a university degree or higher, both substantially above the corresponding figures for the general adult population. The sample is therefore not representative of adults in Buraydah or the Qassim region, and the findings should be generalised with caution. A conventional response rate could not be calculated because the survey link was forwarded onward by administrators and staff.

Eligibility for the present analysis was restricted to participants using Snapchat at least two to four times daily. This restriction was adopted so that the intensity items would be meaningful to respondents, but it truncates the exposure distribution at the lower end and means that the results describe variation in intensity among habitual users rather than the full range from non-use to heavy use. The subgroup also differed from the full project sample in age, gender composition, and overall intensity of social media use, so the findings should not be generalized to the project sample as a whole.

Both exposure and outcomes were measured by self-report, and responses may have been affected by social desirability bias or inaccurate recall of actual patterns of use.

The Snapchat Intensity Scale was adapted from the Facebook Intensity Scale and has not been formally validated in an Arabic-speaking population. Internal consistency in the present sample was good, but factor structure and convergent validity were not examined, and the instrument should be regarded as a working adaptation rather than a validated scale. The high-intensity threshold was derived from this sample and has no external validation.

The cross-sectional design does not permit conclusions about the direction of the observed associations. Residual confounding is also possible, since personality traits, preexisting psychiatric diagnosis, socioeconomic circumstances beyond income, and life stressors were not measured.

Finally, Snapchat usage intensity was not independently associated with symptom scores after adjustment for problematic social media use. The present data cannot determine whether intense engagement with a single platform carries risk distinct from a broader tendency toward problematic use, and the findings should be read as describing Snapchat intensity within that wider context rather than as identifying a platform-specific effect.

## Conclusions

This study found a weak association between the intensity of Snapchat use and symptoms of depression and anxiety among adult Snapchat users in the Qassim region of Saudi Arabia. The association persisted after adjustment for age, marital status, and income, but the effect sizes were small, accounting for less than 10% of the variance in symptom scores.

Causality cannot be inferred from a cross-sectional design, and reverse causation and reciprocal influence remain plausible. The findings suggest that emotional and behavioral engagement may be a relevant dimension of social media use to consider in mental health assessment, although this study does not establish that such engagement is more strongly related to symptoms than duration of use.

Because the association was attenuated by adjustment for problematic social media use, these results are best understood as describing one expression of a broader pattern of engagement rather than a platform-specific risk. Longitudinal designs and studies incorporating validated measures of the proposed mechanisms, including fear of missing out, social comparison, and sleep quality, are needed to clarify these relationships and to inform digital literacy and public health strategies.
